# P-1658. Healthcare Provider Perspectives on Antibiotic Use in Immigrants and Refugees in the United States: A Qualitative Study

**DOI:** 10.1093/ofid/ofae631.1824

**Published:** 2025-01-29

**Authors:** Rachel Croxton, Joseph Ladines-Lim, Timothy Schurz, Timothy Guetterman, Payal K Patel

**Affiliations:** University of Michigan, Ann Arbor, Michigan; University of Michigan, Ann Arbor, Michigan; University of Michigan, Ann Arbor, Michigan; University of Michigan, Ann Arbor, Michigan; Intermountain Healthcare, Salt Lake City, UT

## Abstract

**Background:**

Antimicrobial resistance (AMR) is a global health threat with greater prevalence in refugee populations. Prior work demonstrated that compared to native-born Americans, immigrants/refugees from low- and middle-income countries have lower health literacy about AMR, increasing risk for inappropriate antibiotic use. Healthcare provider (HCP) perceptions of prescribing practices regarding immigrant/refugee populations in the United States (US) is lacking. We aimed to understand US HCP views on antibiotic prescribing practices and barriers that may lead to disparities affecting immigrants/refugees.

**Figure:**
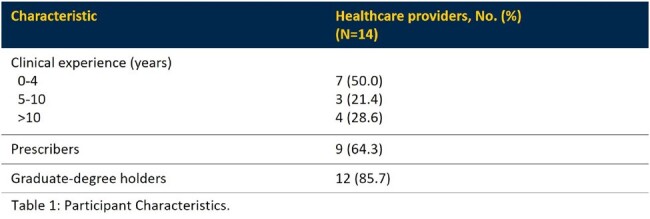

**Methods:**

From September 1, 2023 through April 27, 2024, as a follow up to patient-centered work on antibiotic knowledge in the immigrant/refugee population, we conducted semi-structured qualitative interviews of 14 HCPs, both prescribers (e.g. physicians, advanced practice providers) and non-prescribers (e.g. nurses, medical assistants), from primary care clinics in SE Michigan that care for immigrants/refugees. We also collected participant demographic information. We generated a codebook through an iterative, inductive process using Dedoose software to code interviews and facilitate thematic analysis.

**Figure:**
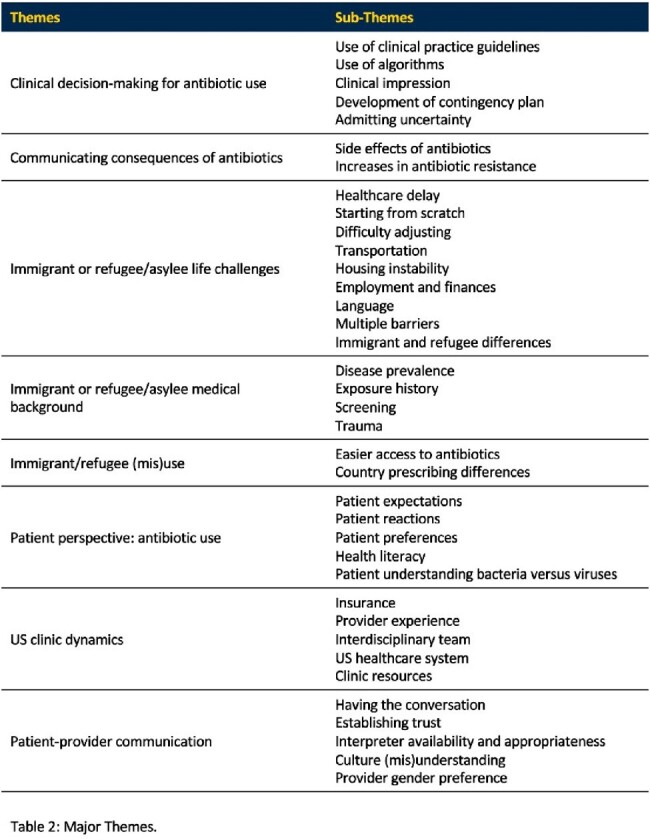

**Results:**

Self-reported proportions of participants’ patient panels comprised of immigrants, refugees, and non-English speakers were 31.0%, 30.1%, and 48.2% , respectively. Most participants were prescribers and graduate-degree holders (Table 1). Eight major themes emerged: Clinical Decision-Making, Discussing Antimicrobial Consequences, Patient Life Challenges, Patient Medical Background, Antibiotic Use Differences, Patient Perspectives, Clinic Dynamics, and Patient-Provider Communication. There was consensus that distinctive challenges exist for appropriate antimicrobial prescribing among immigrant/refugee populations (Table 2).

**Conclusion:**

Findings suggest that immigrant/refugee populations face multiple barriers to care. This population may benefit from an interdisciplinary outpatient team to navigate cultural differences and trauma-informed care. Our findings can inform ambulatory antibiotic stewardship interventions in immigrant/refugee populations.

**Disclosures:**

**All Authors**: No reported disclosures

